# Relationship of Thyroid Hormone Levels to Levels of Polychlorinated Biphenyls, Lead, *p,p*′*-* DDE, and Other Toxicants in Akwesasne Mohawk Youth

**DOI:** 10.1289/ehp.10490

**Published:** 2008-02-25

**Authors:** Lawrence M. Schell, Mia V. Gallo, Melinda Denham, Julia Ravenscroft, Anthony P. DeCaprio, David O. Carpenter

**Affiliations:** 1 Department of Anthropology, University at Albany, State University of New York, Albany, New York, USA; 2 Department of Epidemiology and Biostatistics, School of Public Health, University at Albany, State University of New York, Rensselaer, New York, USA; 3 School of Public Health and Health Sciences, University of Massachusetts, Amherst, Massachusetts, USA; 4 Institute for Health and the Environment, University at Albany, State University of New York, Rensselaer, New York, USA

**Keywords:** adolescents, hexachlorobenzene, mercury, mirex, Mohawk, Native American, PCBs, POPs, *p,p*′-dichlorophenyldichloroethylene, thyroid, thyroid hormones

## Abstract

**Background:**

It is well documented that acute exposure to high levels of persistent organic pollutants, such as polychlorinated biphenyls (PCBs), *p,p*′*-*dichlorophenyldichloroethylene (*p,p*′*-*DDE), and hexachlorobenzene (HCB), can affect human health including thyroid function. Chronic exposure to multiple toxicants is common but difficult to analyze, and most prior studies have focused on adults or newborns, creating a gap in our understanding of multitoxicant effects among adolescents.

**Objective:**

We investigated whether levels of PCBs, *p,p*′*-*DDE, HCB, mirex, lead, and mercury reflecting past chronic exposure are associated with alterations in levels of thyroid-stimulating hormone (TSH), triiodothyronine (T_3_), total thyroxine (TT_4_), and free thyroxine (FT_4_) among older children and adolescents.

**Methods:**

The sample consists of youth from the Akwesasne Mohawk Nation (*n* = 232) who reside in proximity to several industries that have contaminated the local environment. We used multiple regression analysis to examine the effect of PCB groupings, *p,p*′*-*DDE, HCB, lead, and mercury on thyroid hormones after adjusting for sociodemographic covariates and controlling for all other toxicants.

**Results:**

Exposure to PCBs affects the thyroid hormone profile in adolescents. The group of persistent PCBs was positively associated with TSH but inversely related to FT_4_. Nonpersistent PCBs were significantly and negatively related to FT_4_ only. HCB was negatively associated with T_4_, and lead was positively associated with T_3_. Breast-fed adolescents had higher levels of persistent PCBs and *p,p*′*-*DDE but not of nonpersistent PCBs or any other toxicant when compared with non-breast-fed adolescents. Though having lower levels of persistent PCBs and *p,p*′*-*DDE, non-breast-fed adolescents exhibited significant relationships between persistent PCBs and TSH and FT_4_, but breast-fed adolescents did not. It appears that PCBs from breast milk obscure the relationship between prenatal PCB exposure and thyroid function by adding random variation in PCB levels.

**Conclusion:**

Our results demonstrate a reduction in thyroid function in adolescents in relation to their current serum levels of PCBs. These observations are consistent with the hypothesis that pre-natal exposure to PCBs alters thyroid function in a long-lasting manner but does not exclude the possibility that postnatal exposure is influential also.

Exposure to persistent organic pollutants (POPs) such as polychlorinated biphenyls (PCBs), *p,p*′*-*dichlorophenyldichloroethylene (*p,p*′*-*DDE), a metabolite of *p,p*′*-* dichlorodiphenyltrichloroethane (*p,p*′*-*DDT), and hexachlorobenzene (HCB) is a global phenomenon. High levels of exposure to these and other potentially endocrine-disrupting toxicants affect human health, but the full extent of their impact remains an important area of study (Carpenter 2006; Daston et al. 2003; Kimbrough and Krouskas 2003; Koppe et al. 2006; Langer 2005). Experimental animal studies have demonstrated effects of POPs on endocrine system functioning, including thyroid function (Brucker-Davis 1998). These studies typically investigate large, single-toxicant exposures that are uncharacteristic of most human exposure patterns. Studies in humans have found relationships between exposure to specific POPs and lower thyroid hormone levels as well as higher thyroid-stimulating hormone (TSH) levels (Koopman-Esseboom et al. 1994; Nagayama et al. 1998; Osius et al. 1999; Persky et al. 2001; Ribas-Fito et al. 2003; Rylander et al. 2006; Sauer et al. 1994; Wang et al. 2005; Zuurbier et al. 2006). Langer et al. (2005) have demonstrated that PCB exposure results in an increase in thyroid gland volume but an inverse relationship with TSH (2006). However, not all investigators have observed such associations, and as human studies have focused almost entirely on infants and adults (Meeker et al. 2006), the relationship of toxicants to thyroid hormone status in older children and adolescents is not apparent (Hagmar 2003).

Thyroid hormones are essential, regulating metabolism and promoting normal cardiovascular, reproductive, and nervous system functioning (Larsen et al. 2003). They are necessary for normal growth and brain development (Porterfield and Hendrich 1993), and thus may represent a causal link between toxicants and observed effects on somatic growth and cognitive development.

The aim of the current investigation is to assess whether levels of PCBs indicative of a chronic exposure pattern are associated with alterations in levels of TSH, triiodothyronine (T_3_), total thyroxine (TT_4_), and free thyroxine (FT_4_) among older children and adolescents. In addition to congener-specific PCB analyses, we also examined the effects of other common toxicants (*p,p*′*-*DDE, HCB, mirex, lead, and mercury) on thyroid hormone levels.

## Methods

### Setting

The study was conducted with mother–youth pairs who were members of the Akwesasne Mohawk Nation (Akwesasne), which spans the St. Lawrence River with territory in New York State and in Ontario and Quebec, Canada. Industrial development along the St. Lawrence River began in the 1950s and continues today, with several industrial complexes located near Akwesasne. A National Priority Superfund Site (General Motors Central Foundry Division) and two New York State Superfund Sites (Reynolds Metal Company and Aluminum Company of America) are located immediately upstream. In the 1990s, some local animal species were found to have levels of PCBs, *p,p*′-DDE, HCB, and mirex above human consumption tolerance limits set by the U.S. Food and Drug Administration (Forti et al. 1995; Sloan and Jock 1990). In 1986 and 1987, advisories against eating locally caught fish were issued. Local informants have attested to a reduction in consumption of locally caught fish, and studies of PCB levels in breast milk reported a decrease in levels subsequently that is consistent with adherence to the advisories (Fitzgerald et al. 2001). Thus, adolescents born after the fish advisories may have had less post-natal exposure than those born before the advisories, especially exposure to persistent PCBs.

Human health studies at Akwesasne, including an investigation of adolescent development, were prompted by the history of local environmental pollution from the neighboring industrial point sources. Long-standing reliance on locally caught fish and game (prime pathways of exposure) and concerns of community members about the health effects of environmental pollutants for themselves and future generations resulted in a community-based research collaboration.

### Participants

The study began in 1995 and ended in 2000. Study methods, including recruitment and data collection protocols, laboratory analysis methods, and substitution methods for toxicant levels below the laboratory minimum detection limits, are briefly described here [for greater detail, see Schell et al. (2003)]. Akwesasne community members collected all data without prior knowledge of the exposure status of participants. The Institutional Review Board at the University at Albany, State University of New York, approved all study protocols. Informed consent, and assent from minors, was obtained from all participants.

The target population was defined as residents of Mohawk households located in the Akwesasne Mohawk Nation and in neighboring communities within 10 miles of Akwesasne. Mother–youth participant pairs were eligible for the study if they lived in the same household, and if the adolescent was between the ages of 10 and 16.99 years, not a twin, not diagnosed with a psychological or physical impairment, and not diagnosed with fetal alcohol syndrome or effects. Of 294 mother–youth pairs who met the study eligibility requirements and enrolled in the study, 271 continued participation and had blood available for analysis. Of the 271, 19 pairs had missing data for covariates included in the subsequent multiple regression analysis. Of the 252 remaining participants, 7 youth were missing either T_4_ or FT_4_ results. Of those remaining, 13 additional participants were excluded from this analysis because of a change in laboratory methods for the analysis of TSH and thyroid hormones, for a final sample size of 232.

### Blood collection and laboratory analysis

Fasting blood specimens were collected at first rising by trained Mohawk staff and provided material for analysis of six toxicants (lead, mercury, PCBs, *p,p*′-DDE, HCB, and mirex), cholesterol, triglycerides, TSH, and thyroid hormones. All samples were drawn between 1996 and 2000.

PCB and organochlorine pesticide analyses were conducted at the Exposure Assessment Laboratory of the University at Albany. High-resolution, ultratrace, congener-specific analysis was performed by parallel dual-column (splitless injection) gas chromatography with electron capture detection on an Agilent (Santa Clara, CA) 5890 instrument (DeCaprio et al. 2000). This method quantitates up to 83 individual PCB congeners and 18 PCB congeners as pairs or triplets, as well as *p,p*′-DDE, HCB, and mirex. Data were expressed on a whole-weight basis. Analyses of lead and mercury were conducted by Le Centre de Toxicologie due Quebec in Sainte-Foy, Quebec, Canada. Mercury analysis was based on cold-vapor atomic absorption spectrometry using a Pharmacia (Stockholm, Sweden) model 100 mercury monitor. Levels are reported as the sum of organic and inorganic mercury in micrograms per deciliter. Lead was analyzed by Zeeman-corrected graphite furnace atomic absorption spectrometry on a PerkinElmer (Waltham, MA) model 4100ZL instrument.

Assessment of cholesterol, triglycerides, TSH, T_3_, T_4_, and FT_4_ was performed at the Clinical Chemistry and Hematology Laboratory, Wadsworth Center for Laboratories and Research, New York State Department of Health (Albany, NY). The facility is approved by the Clinical Laboratory Improvement Amendments and is a member of the Centers for Disease Control and Prevention (CDC) reference laboratory network for lipid measurements (Myers et al. 2000). Serum lipid concentrations were measured on a Hitachi 911 analyzer (Roche Diagnostics, Indianapolis, IN) using a cholesterol esterase and oxidase/peroxidase method for total cholesterol (Allain et al. 1974) and a glycerol kinase-based procedure that corrects for free glycerol in the specimen (Kohlmeier 1986) for triglycerides. Thyroid hormones were analyzed by ultrasensitive radioimmunoassay using standard methodologies (National Academy of Clinical Biology 2002). Sensitivity for TSH was 0.02 μIU/mL, with a coefficient of variation of < 20%. Reference ranges for clinically normal values were 4.5–12.5 μg/dL and 0.71–1.85 ng/dL for T_4_ and FT_4_, respectively, 85–190 ng/dL for T_3_, and 0.3–5.0 for TSH.

### Pollutants

Values below the method detection limits for lead, mercury, PCBs, and HCB were imputed using the U.S. Environmental Protection Agency recommended method (U.S. EPA 1998) for toxicants with rates of detection of ≥ 50%. This method imputes a value for each datum below the method detection limit based on the method detection limit value, the percentage of observations below the method detection limit, and the mean and variance of the detected observations. No imputation method was used for *p,p*′-DDE because all participants had detectable levels. To correct for skewness and normalize the distributions, lead, mercury, PCBs, *p,p*′-DDE, and HCB were natural log transformed. The 16 PCB congeners detected in > 50% of the sample are included in our analyses for hypothesis testing. PCB congeners were considered individually and in three groups: *a*) all 16 PCB congeners: PCB congeners detected in > 50% of the sample (PCB-50%): International Union of Pure and Applied Chemistry (IUPAC) congeners 52, 70, 74, 84, 87, 95, 99, 101[+ 90], 105, 110, 118, 149[+ 123], 138[+ 164 + 163], 153, 180, 187; *b*) eight persistent PCB congeners (PCB-PER8): IUPAC congeners 74, 99, 105, 118, 138[+ 164 + 163], 153, 180, 187; and *c*) eight nonpersistent congeners (PCB-NON8): IUPAC congeners 101[+ 90], 110, 95, 52, 149[+ 123], 84, 70, 87. Persistent PCBs are congeners with long (years) physiologic half-lives in humans (Brown 1994; Hansen 1998). IUPAC congeners 70 and 87 are classified as nonpersistent based on data presented by Brown (1994). Mirex levels were categorized into three groups because > 50% of the sample had levels below the method detection limit of 0.02 ppb: nondetects (< 0.02 ppb; 54.7%), low detects (0.02–0.03 ppb; 17.2% of the sample), and high detects (0.04–1.17 ppb; 28.0% of the sample).

### Other variables

Additional information was obtained by interview with the mother of the youth, including sociodemographic variables and breast-feeding history, which was recorded as “any” or “none.” If a blood sample could not be analyzed at the laboratory, it was redrawn at a later point, occasionally creating a lag between collection of interview data and blood draw. The variable—time to blood analysis—describes this time difference. However, all biologic material analyzed to determine thyroid hormones and toxicant levels was from blood drawn at a single point in time.

### Statistical analysis

We used multiple regression analysis to examine the effect of each toxicant on thyroid hormones when controlling for all other toxicants, as well as sex, age, triglycerides, cholesterol, breast-feeding, time of day when blood was collected, and duration of time between interview and blood draw. Covariates were chosen on the basis of bivariate associations (*p* < 0.1, *t*-tests, and correlations) with thyroid hormones and/or PCBs. A PCB-by-breast-feeding interaction was also included in multiple regression analyses. Breast-feeding can be a major route of postnatal PCB exposure, and levels of some PCB congeners typically differ by breast-feeding status. Intercorrelations among toxicants were assessed because high intercorrelations can render the interpretation of multiple regression coefficients problematic.

## Results

The mean (± SD) of adolescents’ age was 13.3 ± 1.94 years. The mean triglyceride level was 86.9 mg/dL, and seven participants had triglyceride values over the laboratory reference range of 200 mg/dL. The mean cholesterol measure was 158.5 mg/dL, with 20 participants above the reference range of 200 mg/dL. The mean time of blood collection was 0831± 0101 hours, just 1 min earlier than the median time. The median time between interview and venipuncture was 1.1 days (−0.003 years), but some cases (27 of 232) had blood redrawn well after interview to repeat the laboratory analysis. Thus, there is a difference between mean and median values for this variable. Nearly half (45.7%) of the youth were breast-fed as infants.

Mean levels of T_4_, FT_4_, T_3_, and TSH were within the laboratory reference range (Table 1). Eight participants had TSH levels above the laboratory reference range, and four had T_3_ levels above the normal range. Few participants had T_3_, T_4_, or TSH levels below the laboratory reference range (*n* = 3, 3, and 4, respectively). FT_4_ and T_4_ were significantly lower in youth who had been breast-fed, whereas TSH was significantly higher. T_3_ did not differ.

Lead, mercury, HCB, *p,p*′*-*DDE, and PCB levels of Mohawk adolescents are described in Table 2. Toxicant levels in the study sample were consistent with a pattern of chronic exposure to multiple toxicants. The highest level of lead in the sample was less than half of the CDC action level of 10 μg/dL (CDC 1991). Mercury levels of Mohawk adolescents were at or below background levels of 0.1–0.8 μg/dL that have been reported for the general population (Agency for Toxic Substances and Disease Registry 1999). Only one Mohawk adolescent had a mercury level ≥ 5.8 μg/L, the blood mercury level equivalent to the U.S. EPA reference dose, compared with 5.66% of women of childbearing age in the general population (CDC 2004). PCB levels of adolescents and the proportion of persistent to nonpersistent congeners were consistent with both cumulative and recent exposure [58% of the most common congeners (PCB-50%) were persistent ones] (DeCaprio et al. 2005; Schell et al. 2003).

PCB-50%, PCB-PER8, and *p,p*′*-*DDE were significantly higher in the breast-fed participants (Table 2). When individual PCB congener levels were compared, all persistent congeners, except for PCB-105, had significantly higher values among adolescents who were breast-fed as infants. Levels of all nonpersistent congeners, except for PCB-87, were not significantly different by breast-feeding status. No difference in lead, mercury, or HCB was observed by breast-feeding status. We also considered other factors that could differ between non-breast-fed and breast-fed youth (age, sex, two indices of socioeconomic status, triglycerides, cholesterol, and the youth’s weight, height, and body mass index) and found no significant differences.

We used multivariate regression analyses to examine the effects of different measures of PCB exposure on thyroid hormone and TSH levels while controlling for other toxicants (HCB, *p,p*′*-*DDE, mirex, lead, mercury) and additional covariates [age, sex, cholesterol, triglycerides, time of collection (time of day), time to blood analysis (years), breast-feeding, and breast-feeding by PCB interaction]. Results are presented for two regression analyses using PCB-PER8 as the PCB measure to predict log-transformed TSH (Table 3) and FT_4_ (Table 4). PCB-PER8 was positively associated with TSH. A breast-feeding-by-PCB-PER8 interaction was statistically significant. From this regression analysis, we calculated the effect of PCB-PER8 on TSH and FT_4_ in both the breast-fed and non-breast-fed adolescents while holding all other model variables constant at their respective means. As PCB-PER8 levels rise from 0.204 ppb at the 5th percentile to 0.871 ppb at the 95th percentile, estimated TSH levels increase by 1.51 μIU/mL among adolescents who were not breast-fed as infants. Among adolescents who were breast-fed as infants, TSH levels are not significantly related to PCB-PER8; at the 95th percentile of PCB-PER8, the estimated TSH level is 2.8 μIU/mL compared with 3 μIU/mL at the 50th percentile level. Repeating the analyses without the individuals with TSH, T4, T3 levels outside the laboratory range produced results, including those for the interaction effect, that were unchanged for effects of PCB-PER8, and only slightly different for effects of PCB-50% and lead. The beta coefficient estimating the relationship between PCB50% and T_4_ increased trivially from −0.06 to −0.065, with a change in *p*-value from 0.057 to 0.033. The effect of lead in the model was attenuated, and *p*-values increased to just above 0.05.

Table 4 shows a negative effect of PCB- PER8 on FT_4_. The PCB-PER8 by breast-feeding interaction term was positive but not statistically significant. Applying this model to the non-breast-fed group, predicted FT_4_ levels decrease from 3.1 ng/dL at the 5th percentile of PCB-PER8 to 2.9 ng/dL at the 50th percentile and to 2.7 ng/dL at the 95th percentile. Among breast-fed adolescents, FT_4_ was essentially unchanged with increasing PCB-PER8.

PCB-NON8 was not significantly related to TSH but was significantly and negatively related to FT_4_. Because congener groups were related to either TSH, FT_4_, or both, the constituent congeners were tested individually (Table 5). Of the persistent congeners, 118, 138[+ 163 + 164], and 153 were positively associated with TSH levels, and only one nonpersistent congener (110) showed a relationship with TSH. Two persistent and six nonpersistent PCB congeners were negatively associated with FT_4_, as were all three summary PCB measures.

HCB was negatively associated with T_4_, and lead was positively associated with T_3_ (results not shown in table). Results for HCB and lead were consistent regardless of which measure of PCB burden was included in the multivariate model.

On examination of the interrelationships among toxicants, *p,p*′-DDE was found to be positively correlated with PCB-50%, PCB-PER8, and HCB, with coefficients of 0.46, 0.60, and 0.39, respectively. Collinearity among toxicants was not considered an analytical complication, because the largest coefficient was well below 0.80. To ensure that collinearity was not influential, the regression analysis was performed with models trimmed of covariates that were not significant (*p* < 0.1) in any single model (time of blood collection, *p,p*′*-*DDE, mirex nondetects vs. detects). Trimmed models were little changed (< 20% change in beta coefficients, and the same PCB and thyroid effects were significant at *p* < 0.05; results not shown) when compared with full models that included time of blood collection, *p,p*′*-*DDE, and mirex nondetects versus detects.

## Discussion

The most striking observation in this study is the demonstration of a significant positive relationship between serum PCB levels and TSH in adolescents who were not breast-fed, and the lack of such a relationship in adolescents who were breast-fed—despite the higher PCB levels found in breast-fed adolescents. There was also a negative but less significant relationship between serum PCB levels and FT_4_ in non-breast-fed, but not in breast-fed, adolescents.

Breast-feeding has been shown to be the largest postnatal source of PCB burden in chronically exposed populations (Chao et al. 2004; Lackman et al. 2004; Patandin et al. 1999). One would therefore expect PCB effects to be greater in those youth with higher PCB levels, but this is not the case here. The non-breast-fed youth displayed evidence of stronger associations between PCB levels and TSH and FT_4_ than the breast-fed group with higher PCB levels. It is possible that breast-feeding has a beneficial effect, reducing the impact of prenatal PCB exposure on measures of thyroid function. Studies have documented the benefits of breast-feeding on development, mortality, and morbidity (Anderson et al. 1999; Howie et al. 1990; Kramer et al. 2001; Singhal et al. 2001; WHO Collaborative Study Team on the Role of Breastfeeding on the Prevention of Infant Mortality 2000; Wilson et al. 1998). However, the suggestion that breast-feeding is protective of PCB-induced alterations of thyroid function is not concordant with the higher levels of PCBs in breast-fed adolescents and the expectation of a dose–response relationship.

A more likely explanation for our observations is that prenatal exposure to PCBs alters thyroid function to a greater degree than early postnatal exposure or exposure during childhood and adolescence. The notion of a critical window is consistent with findings on organochlorine exposure and brain development. In newborn mice where brain development is in a critical period, exposure to persistent organochlorines, such as PCBs, has been shown to cause severe, sometimes irreversible, brain disruption (Eriksson 1997; Eriksson et al. 2002). If the effect of PCBs on thyroid function is sensitive to the timing of the exposure also, an effect of PCBs that occurs only with prenatal exposure might be observable only among adolescents who experienced little postnatal exposure to PCBs. For non-breast-fed adolescents, PCB levels would be expected to more closely reflect prenatal exposure. Breast-feeding exposes children to a comparatively large dose of PCBs and is a major source of overall PCB body burden (Ayotte et al. 2003; Lackman et al. 2004; Lanting et al. 1998). Thus, breast-feeding may add PCBs that obscure the relationship between prenatal PCB exposure and thyroid function by adding random variation in PCB levels, rather than protecting the thyroid from the disruptive influence of PCBs. Breast-feeding is not associated with additional risk to thyroid disruption, despite the higher levels of PCBs resulting from breast-feeding.

There are a number of reasons to suspect that prenatal PCB exposure might alter thyroid function later in life. PCBs disturb differentiation of normal human neural progenitor cells, a thyroid hormone–dependent process (Fritsche et al. 2005); inhibit thyroid hormone–dependent extension of dendrites (Kimura-Kuroda et al. 2005); and cause a decrease in pituitary and thyroid responses to thyrotropin-releasing hormone stimulation (Khan and Hansen 2003). Gauger et al. (2004) have reported that PCBs have direct actions on several thyroid hormone–responsive genes in the fetal rat brain and increase the expression of neuroendocrine-specific protein A, RC3/neurogranin, and Oct-1. These actions occur independently of the reduced circulating levels of T_3_ and T_4_ in the dam. Miyazaki et al. (2004) found that PCBs suppress thyroid receptor–mediated transcription and suggested that this is particularly the case in the developing nervous system. Thus, there are several possible mechanisms whereby PCB exposure during development might alter thyroid function permanently.

Prenatal exposure to PCBs also causes a greater and more persistent alteration of other organ systems than does postnatal exposure. In children, decrements in neurobehavioral function resulting from PCB and dioxin exposure are primarily a result of prenatal exposure (Lai et al. 2001; Vreugdenhil et al. 2002). Prenatal exposure to PCBs and dibenzofurans has been found to alter semen quality (Guo et al. 2000) and sperm function (Hsu et al. 2003) in adult humans and alter fertility in adult rats (Kuriyama and Chahoud 2004).

Our results show that not all PCB congeners have similar effects. We observed statistically significant, positive associations between TSH levels and PCB congeners 110, 118, 138[+ 163 + 164], 153, as well as with two PCB groupings (PCB-50%, PCB-PER8). Of the four individual congeners/triplets, three are persistent, three are di-*ortho* congeners, and all four are highly chlorinated. Negative associations were observed between FT_4_ levels and three congener groupings (PCB-50%, PCB-PER8, PCB-NON8), as well as congeners 52, 70, 84, 87, 101[+ 90], 138[+ 163 + 164], 149[+ 123], and 153. Six of these eight congeners are highly chlorinated, and five are di-*ortho* congeners. Only congener 87 was associated with T_4_ levels, and none of the tested PCB congeners or groupings was associated with T_3_ levels. The results presented here on the relationships of PCBs to TSH, FT_4_, and T_3_ are consistent with a preliminary report on a sample of 113 Mohawk adolescents who participated in this study (Schell et al. 2002), and with a recent report showing an increasing incidence rate of hypothyroidism among patients using the St. Regis Mohawk Health Service between 1992 and 1995 (Negoita et al. 2001).

The positive association observed here between PCBs and TSH, and the negative association between PCBs and thyroid hormones is supported by the literature. PCBs (as individual congeners or mixtures) have repeatedly been shown to alter thyroid function and hormone levels in experimental animals, including thyroid-hormone suppression and cell-mediated immunomodulation (Brouwer et al. 1998; Hallgren et al. 2001; Kato et al. 1998; Kuriyama et al. 2003; Li and Hansen 1996a, 1996b; McNabb and Fox 2003; Morse et al. 1993; Ness et al. 1993; Seo et al. 1995; Smits et al. 2002). *In utero* exposure of rats produces depressed plasma T_4_ levels in late gestation and in newborns (Morse et al. 1993; Ness et al. 1993; Seo et al. 1995), and prepubertal exposure can depress serum T_4_ levels in rats as well (Li and Hansen 1996a, 1996b). Studies of PCB-thyroid effects in humans have been less consistent. In men and women who consumed fish from the Great Lakes, serum PCBs were associated with lower T_4_ levels. Inconsistent associations were found with TSH and PCBs (Persky et al. 2001). Using data from the National Health and Nutrition Examination Survey (NHANES) (1999–2002), Turyk et al. (2007) found PCBs to be positively related to TSH among older women, yet inversely associated among older men. Additionally, a negative relationship of TEQ to T_4_ was seen in both men and women, with a stronger correlation among women. No associations were reported for younger NHANES participants (Turyk et al. 2007). A study of men between 20 and 64 years of age found inverse relationships between T_3_ and two persistent PCB congeners (138, 153), the sum of PCBs, and HCB (Meeker et al. 2007). In the Dutch multicenter study of newborns, higher PCB and dioxin levels in breast milk were positively associated with TSH levels and negatively associated with FT_4_ levels (Koopman-Esseboom et al. 1994; Sauer et al. 1994). One-year-old Japanese infants who were breast-fed had lower T_3_ and T_4_ levels at higher levels of polychlorinated dibenzo-*p*-dioxins and dibenzofurans and coplanar PCBs in mothers’ milk, although TSH levels were unrelated (Nagayama et al. 1998). Positive associations between congener 118 and TSH levels have been reported in 320 German schoolchildren between 7 and 10 years of age (Osius et al. 1999), and 98 newborns in Spain (Ribas-Fito et al. 2003), as well as in the present study. A longitudinal study of 38 breast-fed infants in the Netherlands reported a positive association between dioxin concentration in breast milk and TSH levels at 11 weeks (Pluim et al. 1992, 1993). However, Pluim and coworkers (1992 (1993) also observed higher T_4_ levels at birth, 1 week, and 11 weeks, contrary to results reported here as well as by others described above. Negative associations between serum PCB levels and FT_4_ have been reported in neonates from coastal communities in Quebec (Sandau et al. 2002) and adults from a Spanish village (Sala et al. 2001), whereas in an adult population from Quebec there was a negative relationship between levels of three PCB congeners (138, 153, and 180) and T_3_ but not with TSH or FT_4_ (Takser et al. 2005).

PCBs have long been suspected to affect thyroid hormone signaling because of structural similarities between PCBs and thyroid hormones (Porterfield 1994). Evidence suggests that two plausible mechanisms may be responsible for the PCB-induced reduction in thyroid hormones, particularly T_4_: disruption of thyroid hormone transport (Cheek et al. 1999; Darnerud et al. 1996) and induction of hepatic metabolism (Morse et al. 1993; Zhou et al. 2001). Results presented here are consistent with either mechanism of action. In the former mechanism, thyroid hormones are bound and transported primarily by two plasma proteins in humans, transthyretin (TTR) and thyroid-binding globulin (TBG) (Larsen et al. 2003). TTR is more likely to be involved as the transport protein in humans, because hydroxylated PCBs have greater affinities for TTR than does T_4_ (Cheek et al. 1999; Lans et al. 1993, 1994; Rickenbacher et al. 1986). In contrast, few hydroxylated or unmetabolized PCBs bind TBG (Lans et al. 1993, 1994). In one study, after PCB exposure, the degree of thyroid hormone reduction in rats coincided with binding of T_4_ to the plasma thyroid hormone transporter TTR, suggesting that PCB-induced T_4_ reductions were attributed primarily to disturbed transport (Hallgren and Darnerud 2002). The second mechanism that has been investigated is induction of hepatic metabolism. Several studies have suggested that PCB-induced T_4_ reductions result from increased metabolism of the hepatic microsomal enzyme uridine diphosphoglucuronosyl transferase (UDPGT). This induction is primarily the result of aryl hydrocarbon receptor activation by dioxin-like PCB congeners (Barter and Klaassen 1992, 1994; Beetstra et al. 1991; Morse et al. 1993; Van Birgelen et al. 1995; Zhou et al. 2001). UDPGT catalyzes glucuronidation of T_4_ and consequently increases biliary excretion of T_4_ (Bastomsky 1974). However, others suggest a moderate or nonsignificant effect of UDPGT induction on PCB-mediated decreases in T_4_ levels (Hallgren and Darnerud 2002; Hallgren et al. 2001). Because of insufficient specimen availability, we could not obtain data for the most potent dioxin-like congeners (CBs 126 and 169), which are typically present in human serum at levels two or three orders of magnitude lower than the most prevalent congeners. Therefore, we could not employ a dioxin-like TEQ approach (Van den et al. 2006) that may have provided additional support for the latter mechanism.

We also observed a negative association of HCB with T_4_ and a positive association between lead and T_3_. The negative association of HCB with T_4_ levels among Akwesasne adolescents is consistent with the literature. Numerous animal studies have demonstrated HCB-induced hypothyroidism, with T_4_ levels being particularly sensitive in rats (Alvarez et al. 2005; Foster et al. 1993; Kleiman de Pisarev et al. 1990, 1995; Rozman et al. 1986; van Raaij et al. 1993). Lower T_4_ levels associated with HCB exposure may be attributed to peripheral disposition of T_4_ (Kleiman de Pisarev et al. 1989) or increased hepatic T_4_ metabolism (Kleiman de Pisarev et al. 1990). It has also been suggested that HCB affects the thyroid in rats via its metabolites, particularly the main metabolite pentachlorophenol, an effective competitor for T_4_ binding sites (van Raaij et al. 1991a, 1991b). In Turkey, 37% of patients with HCB-induced porphyria also developed enlarged thyroid glands (Gocmen et al. 1986). In a highly exposed, rural population residing in Catalonia, Spain, HCB levels were associated with lower T_4_ levels (Sala et al. 2001). However, no relationship was observed between HCB and TSH levels in 98 newborns born in a highly HCB-polluted area (Ribas-Fito et al. 2003).

Although animal studies have shown lead to inhibit thyroid function and reduce circulating levels of T_3_ and T_4_ (Sandstead 1967; Shrivastava et al. 1987; Singh and Dhawan 1999), studies of thyroid functioning in humans have yielded mixed results. Some report no relationship (Erfurth et al. 2001; Gennart et al. 1992; Schumacher et al. 1998), whereas others report depressed thyroid hormone levels and/or function (Liang et al. 2003; Robins et al. 1983; Sandstead et al. 1969). It is also possible that lead may have different effects on thyroid hormones at different levels of exposure. In a study among occupationally exposed men, lead was positively associated with T_3_, T_4_, FT_4_, and TSH among workers with blood lead levels from 8 to 50 μg/dL and negatively associated with T_3_ and T_4_ when blood lead levels exceeded 50 μg/dL (Lopez et al. 2000). Directional differences in reported effects may stem from different levels and durations of exposure, and may explain the positive association with T_3_ observed here among Akwesasne adolescents.

Toxicant levels in Akwesasne youth (Schell et al. 2003) are somewhat lower than reported levels in older children and adolescents from several other studies (Karmaus et al. 2001; Mazhitova et al. 1998; Nawrot et al. 2002; Osius et al. 1999; Staessen et al. 2001). These populations with higher toxicant levels typically involve acute or known, well-defined sources of exposure. This suggests that toxicant body burdens reported here and, consequently, associations observed with thyroid hormones, may be present in other populations without large or acute exposures. In addition, it is unlikely that iodine insufficiency is responsible for these results. Sodium intake estimated by a semiquantitative food frequency questionnaire (Block et al. 1990, 1992; National Cancer Institute 1999) was more than sufficient to provide the recommended daily intake of iodine, assuming conservatively that only one third of the consumed sodium was iodized. An alternative explanation for thyroid effects in this sample is exposure to fluoride, a thyrotoxicant (National Research Council 2006). In 1980, cattle on Cornwall Island within the reservation and immediately downwind from an aluminum plant exhibited fluorosis despite a fluoride level in forage well below the tolerance level set by the National Academy of Sciences (Crissman et al. 1980). Human populations were exposed to fluoride through locally grown fruits and vegetables rather than from fish, the main route of PCB exposure. There was little evidence of effects on thyroid function: Men and women in the high fluoride group did not differ from those in a low exposure group in terms of T_4_; high fluoride women had higher TSH, whereas the men had a significantly lower TSH level (Selikoff et al. 1983) than those in the low fluoride group.

In summary, our results demonstrate a reduction in thyroid function in adolescents in relation to their serum levels of PCBs, but demonstrate that this relationship is much stronger in adolescents who were not breast-fed, even though breast-fed adolescents have higher serum PCB levels. These observations are consistent with the hypothesis that pre-natal exposure to PCBs alters thyroid function in a long-lasting manner.

## Figures and Tables

**Table 1:** Thyroid hormone levels among Akwesasne Mohawk youth (*n* = 232).

			Percent outside range	
Hormones	Reference range^a^	Mean ± SD	Below	Above
T_4_	4.5–12.5 μg/dL	7.1 ± 1.34	1.3	0
FT_4_	0.71–1.85 ng/dL	1.1 ± 0.16	0	0
T_3_	85–190 ng/dL	138.1 ± 25.93	1.3	1.7
TSH	0.3–5.0 μIU/mL	2.5 ± 1.46	1.7	3.5

a Reference range provided by the Clinical Chemistry and Hematology Laboratory, Wadsworth Center for Laboratories and Research, New York State Department of Health (unpublished data).

**Table 2:** Toxicant levels of Akwesasne Mohawk adolescents: breast-fed and non-breast-fed (*n* = 232).

	Non-breast-fed (*n* = 126)				Breast-fed (*n* = 106)				
Toxicant	GM	Median	SD	Max	GM	Median	SD	Max	*t*
Total PCBs (ppb)									
Zero substitution^a^	0.71	0.68	0.668	3.48	0.95	0.95	0.806	4.52	−2.7**
MDL substitution^b^	1.53	1.41	0.557	3.79	1.75	1.67	0.695	4.74	−2.9**
MDL substitution^c^	2.27	2.14	0.452	4.16	2.47	2.39	0.589	4.96	−3.1**
PCB-50% (ppb)^d^,^e^	0.59	0.55	0.272	1.66	0.77	0.77	0.405	2.36	−4.7**
PCB-PER8 (ppb)^d^,^f^	0.34	0.32	0.168	1.44	0.47	0.46	0.259	1.38	−6.0**
PCB-NON8 (ppb)^d^,^g^	0.25	0.23	0.148	0.94	0.28	0.25	0.203	1.33	−1.7
Persistent PCB congeners (ppb)^d^									
74	0.02	0.02	0.010	0.09	0.03	0.03	0.029	0.22	−5.9**
99	0.04	0.04	0.021	0.13	0.05	0.06	0.034	0.21	−4.3**
105	0.02	0.02	0.011	0.06	0.02	0.02	0.019	0.13	−1.6
118	0.06	0.06	0.029	0.16	0.07	0.07	0.050	0.28	−3.2**
138[+ 163 = 164]	0.06	0.07	0.041	0.34	0.08	0.08	0.056	0.28	−3.5**
153	0.07	0.07	0.047	0.39	0.11	0.11	0.107	0.98	−5.7**
180	0.03	0.03	0.038	0.27	0.05	0.05	0.054	0.35	−4.9**
187	0.02	0.01	0.013	0.13	0.02	0.02	0.014	0.08	−4.3**
Nonpersistent PCB congeners (ppb)^d^									
52	0.03	0.02	0.027	0.16	0.03	0.02	0.029	0.14	−0.7
70	0.02	0.02	0.015	0.11	0.02	0.02	0.019	0.08	−1.5
84	0.02	0.02	0.007	0.05	0.02	0.02	0.010	0.08	−1.3
87	0.03	0.04	0.021	0.13	0.04	0.04	0.027	0.16	−2.3*
95	0.02	0.02	0.014	0.08	0.03	0.02	0.023	0.15	−1.5
101[+ 90]	0.05	0.04	0.034	0.20	0.05	0.05	0.048	0.30	−1.6
110	0.05	0.05	0.033	0.16	0.06	0.06	0.049	0.34	−1.6
149[+ 123]	0.02	0.02	0.016	0.08	0.02	0.02	0.021	0.12	−0.7
*p,p*′-DDE (ppb)	0.31	0.31	0.163	1.23	0.45	0.42	0.416	2.93	−5.9**
HCB (ppb)^d^	0.03	0.03	0.022	0.19	0.04	0.04	0.017	0.13	−1.6
Blood lead (μg/dL)^d^	0.80	1.30	0.965	4.80	0.76	1.45	0.907	3.50	0.3
Mercury (μg/dL)^d^	0.09	0.10	0.093	0.58	0.09	0.08	0.096	0.52	0.6

Abbreviations: GM, geometric mean; Max, maximum; MDL, method detection limit.

a Values below the MDL were substituted with zero.

b Values below the MDL were substituted with one half the MDL.

c Values below the MDL were substituted with the MDL.

d Values below the MDL were substituted with values calculated following the U.S. EPA recommended method.

e Congeners with ≥ 50% detection rate. IUPAC congeners: 52, 70, 74, 84, 87, 95, 99, 101[+90], 105, 110, 118, 149[+ 123], 138[+ 164 + 163], 153, 180, 187.

f Sum of eight persistent PCB congeners with ≥ 50% detection rate. IUPAC congeners: 74, 99, 105, 118, 138[+ 164 + 163], 153, 180, 187.

g Sum of eight nonpersistent PCB congeners with ≥ 50% detection rate. IUPAC congeners: 52, 70, 84, 87, 95, 101[+ 90], 110, 149[+ 123].

* *p* < 0.05.

** *p* < 0.01.

**Table 3:** Multivariate regression analysis predicting TSH levels (μIU/mL)^a^ of Akwesasne Mohawk adolescents (*n* = 232).

Characteristic	β	SE	Std β	*t*	*p*-Value
Constant	1.062	0.674		1.576	0.117
Age (years)	0.026	0.021	0.082	1.250	0.213
Sex (male/female)	−0.187	0.080	−0.150	−2.326	0.021
Breast-feeding (any/none)	−0.202	0.203	−0.162	−0.993	0.322
Time of blood collection	−0.000	0.000	−0.015	−0.230	0.818
Time to blood analysis (years)	−0.065	0.040	−0.103	−1.639	0.103
Cholesterol (mg/dL)	−0.001	0.002	−0.055	−0.778	0.438
Triglycerides (mg/dL)	0.003	0.001	0.234	3.323	0.001
HCB (ppb)^a^	0.084	0.106	0.055	0.798	0.426
*p,p*′-DDE (ppb)^a^	−0.076	0.109	−0.060	−0.695	0.488
Blood lead (μg/dL)^a^	−0.017	0.029	−0.036	−0.562	0.575
Mercury (μg/dL)^a^	−0.026	0.053	−0.031	−0.485	0.628
Mirex (nondetects vs. detects)	−0.104	0.108	−0.083	−0.960	0.338
Mirex (high detects vs. low and nondetects)	−0.219	0.119	−0.158	−1.840	0.067
PCB-PER8 (ppb)^a^	0.431	0.149	0.309	2.899	0.004
PCB-PER8 × breast-feeding	−0.505	0.192	−0.396	−2.626	0.009

Std, standardized.

a Natural log transformed.

**Table 4:** Multivariate regression analysis predicting FT_4_ levels (ng/dL) of Akwesasne Mohawk adolescents (*n* = 232).

Characteristic	β	SE	Std β	*t*	*p*-Value
Constant	1.009	0.183		5.501	0.000
Age (years)	−0.007	0.006	−0.085	−1.237	0.217
Sex (male/female)	0.009	0.022	0.028	0.414	0.680
Breast-feeding (any/none)	0.025	0.055	0.077	0.451	0.652
Time of blood collection	−0.000	0.000	−0.052	−0.786	0.433
Time to blood analysis (years)	0.019	0.011	0.118	1.793	0.074
Cholesterol (mg/dL)	0.000	0.000	0.085	1.156	0.249
Triglycerides (mg/dL)	0.000	0.000	−0.068	−0.927	0.355
HCB (ppb)^a^	−0.027	0.029	−0.067	−0.935	0.351
*p,p*′-DDE (ppb)^a^	−0.003	0.030	−0.008	−0.092	0.926
Blood lead (μg/dL)^a^	0.001	0.008	0.010	0.145	0.885
Mercury (μg/dL)^a^	0.007	0.014	0.033	0.501	0.617
Mirex (nondetects vs. detects)	−0.010	0.029	−0.030	−0.330	0.741
Mirex (high detects vs. low and nondetects)	−0.016	0.032	−0.046	−0.508	0.612
PCB-PER8 (ppb)^a^	−0.099	0.040	−0.272	−2.443	0.015
PCB-PER8 × breast-feeding	0.066	0.052	0.198	1.262	0.208

Std, standardized.

a Natural log transformed.

**Table 5:** Association between different measures of PCBs, each entered individually in multiple regression analyses,^a^ and thyroid hormones and TSH (*n* = 232).

	Standardized β			
PCBs (ppb)^b^,^c^	TSH^b^	FT_4_	T_4_	T_3_
PCB-50%^d^	0.238*	−0.269**	−0.134	−0.053
PCB-PER8^e^	0.309**	−0.272*	−0.121	−0.020
PCB-NON8^f^	0.131	−0.242*	−0.133	−0.061
Persistent PCB IUPAC congeners				
74	0.145	−0.229	−0.111	0.005
99	0.117	−0.173	−0.007	−0.011
105	0.014	−0.022	−0.040	−0.047
118	0.267**	−0.191	−0.049	−0.026
138[+ 163 + 164]	0.349**	−0.234*	−0.116	−0.119
153	0.285**	−0.324**	−0.158	−0.037
180	0.193	−0.125	−0.067	0.037
187	0.164	−0.124	−0.078	−0.033
Nonpersistent PCB IUPAC congeners				
52	0.056	−0.191*	−0.083	−0.100
70	0.121	−0.236*	−0.119	−0.091
84	0.062	−0.226*	−0.120	−0.011
87	0.150	−0.267**	−0.189*	−0.028
95	0.014	−0.196	−0.141	−0.055
101[+ 90]	0.103	−0.214*	−0.095	−0.014
110	0.186*	−0.167	−0.111	−0.070
149[+ 123]	0.176	−0.248*	−0.073	−0.018

a Controlling for age, sex, time of day of collection, time to blood analysis, breast-feeding (any/none), cholesterol, triglycerides, breast-feeding × PCBs, lead, mercury, HCB, DDE, and mirex.

b Log transformed.

c Values below the method detection limit were substituted with values calculated following the U.S. EPA recommended method.

d Congeners with ≥ 50% detection rate. IUPAC congeners: 52, 70, 74, 84, 87, 95, 99, 101[+ 90], 105, 110, 118, 149[+ 123], 138[+164+163], 153, 180, 187.

e Sum of eight persistent PCB congeners with ≥ 50% detection rate. IUPAC congeners: 74, 99, 105, 118, 138[+164+163], 153, 180, 187.

f Sum of eight nonpersistent PCB congeners with ≥ 50% detection rate. IUPAC congeners: 101[+ 90], 110, 95, 52, 149[+123], 84, 70, 87.

* *p* < 0.05.

** *p* < 0.01.
